# Biochemical Effects of Natural and Nanoparticle Fish and Algal Oils in Gilt Pregnancy Diets on Base Excision Repair Enzymes in Newborn Piglets—Socioeconomic Implications for Regional Pig Farming—Preliminary Results

**DOI:** 10.3390/ijms262110676

**Published:** 2025-11-02

**Authors:** Paweł Kowalczyk, Monika Sobol, Joanna Makulska, Andrzej Węglarz, Apoloniusz Kurylczyk, Mateusz Schabikowski, Grzegorz Skiba

**Affiliations:** 1 Department of Animal Nutrition, The Kielanowski Institute of Animal Physiology and Nutrition, Polish Academy of Sciences, Instytucka 3, 05-110 Jabłonna, Poland; m.sobol@ifzz.pl; 2 Department of Genetics, Breeding and Animal Ethology, Hugo Kołłątaj University of Agriculture in Krakow, Ave. A.Mickiewicza 21, 31-120 Krakow, Poland; joanna.makulska@urk.edu.pl (J.M.); andrzej.weglarz@urk.edu.pl (A.W.); 3 Institute of Spatial Management and Socio-Economic Geography, University of Szczecin, Ul. Papieża Jana Pawłą II 22a, 70-453 Szczecin, Poland; apoloniusz.kurylczyk@usz.edu.pl; 4 Institute of Nuclear Physics, Polish Academy of Sciences, Radzikowskiego 152, 31-342 Krakow, Poland; mateusz.schabikowski@ifj.edu.pl

**Keywords:** farm animal, livestock, maternal programming, fish oil, algal oil, nanoparticles, newborn piglets, oxidative stress, DNA repair, nutritional innovation

## Abstract

Base excision repair (BER) is an important mechanism for maintaining genomic integrity and preventing DNA damage and mutations induced by oxidative stress. This study aimed to examine the relationship between oxidative stress and BER activity in newborn piglets by supplementing their mothers’ diets during pregnancy with long-chain n-3 polyunsaturated fatty acids (PUFAs) from algal and fish oils, provided either in natural form or as nanoparticles. BER enzyme activity was assessed using a nicking assay, and their gene expression levels by RT-qPCR in the livers of pregnant gilts and their offspring. Preliminary results indicated that maternal supplementation with oils rich in long-chain n-3 PUFAs significantly reduced (by 32%) BER capacity in the livers of their offspring. A corresponding decrease in mRNA expression of BER genes (*TDG*, *MPG*, *OGG1*) was observed in piglets from gilts receiving fish and algal oil supplements. Maternal supplementation with long-chain n-3 PUFAs may protect foetuses and neonates against oxidative stress, reducing DNA damage and enhancing genomic stability, which could positively influence early postnatal growth. The observed reduction in BER enzyme activity in newborn piglets likely reflected improved DNA integrity, and natural oil forms appeared more effective than their nanoparticle formulations. Disparities in socioeconomic areas related to access to functional foods with health-promoting properties highlight the importance of targeted strategies that integrate local systems and promote nutritional equity.

## 1. Introduction

Both human and animal bodies are constantly exposed to various environmental chemicals that can damage cellular components, including DNA. Unrepaired DNA lesions may accumulate, leading to persistent mutations and contributing to age-related degenerative diseases [1,2,3,4,5,6,7]. One of the major DNA repair pathways is the base excision repair (BER) system, which primarily removes single nucleotide damage caused by oxidation. The BER process initiates with the recognition and excision of the damaged base by a specific DNA glycosylase. Subsequent steps involve cleavage at the abasic site by AP endonuclease, processing by phosphodiesterase, gap filling by DNA polymerase, and final sealing by DNA ligase. This pathway is critical for maintaining genome integrity and preventing mutations that could lead to the development of diseases, including cancer [8,9,10,11,12,13,14]. Maintaining the balance between oxidants and antioxidants is essential for mitigating oxidative stress, which is a common mechanism leading to genomic DNA damage, particularly in organs such as the liver [15,16,17,18,19,20,21,22].

Unsaturated omega-3 fatty acids, especially long-chain n-3 PUFAs, play an irreplaceable role in the proper growth and functioning of many bodily systems. Regular consumption of dietary supplements containing these fatty acids has been shown to significantly improve cardiovascular function [23,24,25,26,27,28] and support central nervous system activity [17] and outcomes in certain neurological and mental disorders [29,30,31,32,33]. Fish and algal oils, which are rich in long-chain n-3 PUFAs such as eicosapentaenoic acid (EPA) and docosahexaenoic acid (DHA) are considered valuable nutritional supplements in countering oxidative stress due to their antioxidant and anti-inflammatory properties [34,35,36,37]. Most studies indicate that regular consumption of long-chain n-3 PUFAs increases endogenous antioxidant and anti-inflammatory molecules and reduces lipid peroxidation [38,39,40,41,42]. Specific effects include increased total antioxidant capacity (TAC) and glutathione peroxidase (GPx) activity, along with decreased malondialdehyde (MDA) content, a marker of lipid peroxidation and oxidative stress [13]. Moreover, dietary supplementation with fats rich in omega-3 PUFAs reduces the concentration of pro-inflammatory prostaglandins (e.g., PGE2) and increases the synthesis of those with anti-inflammatory properties (e.g., PGE3) [2]. Maternal nutrition during pregnancy and/or lactation is known to directly influence foetal and neonatal development, and may exert long-term consequences for postnatal growth [5]. Evidence also suggests that maternal fatty acid intake affects epigenetic modifications in the offspring and regulates gene expression in fatty acid metabolic pathways [3,42]. However, the role of long-chain n-3 PUFAs in preventing foetal/neonatal oxidative stress through maternal nutrition remains less understood and not all studies have reported positive outcomes [16,17,43,44,45,46]. Therefore, this trial investigated whether supplementation of long-chain n-3 PUFAs (EPA and DHA from fish and algal oils) in pregnant sows would affect DNA base damage and BER enzyme activity in their newborn piglets [47,48,49]. It was hypothesised that maternal intake of omega-3–rich fats would protect foetuses and neonates from oxidative stress-induced DNA damage and modulate the activity of the BER system. An in vivo model using newborn piglets was selected due to their physiological similarities to humans, including organ morphology and function, DNA damage repair mechanisms, and metabolic rate [17,18,50,51]. Like human infants, neonatal piglets have underdeveloped immune systems and are highly susceptible to oxidative stress [19,20,21,22,52,53,54,55,56]. Thus, the aim of the study was to quantify genomic DNA damage in the liver, specifically, the levels of ethenoadenine (εA), ethenocytosine (εC), and 8-oxoguanine (8oxoG), as well as the mRNA expression of key DNA repair enzymes, such as thymine-DNA glycosylase (TDG), N-methylpurine DNA glycosylase (MPG), and 8-oxoguanine glycosylase (OGG1). Measurements were performed in newborn piglets from mothers supplemented during pregnancy with natural or nano-encapsulated fish and algal oils [28,30,51,56,57].

It was assumed that the use of nanoparticles of algal and fish oils (their encapsulation) [28,56] in the diet of pregnant gilts, owing to their enhanced bioavailability and absorption, would provide greater protection than natural oil forms against oxidative stress-induced DNA damage and BER pathway impairment in both pregnant gilts and their offspring [17,24,46,51,54,55,56,57,58,59,60,61,62,63,64,65,66,67,68,69,70,71,72,73,74,75,76,77,78,79,80,81,82,83,84,85,86,87,88,89,90,91,92,93,94,95,96,97].

From an economic and social perspective, innovations in the supplementation of long-chain n-3 polyunsaturated fatty acids (PUFAs) in farm animals have an impact on the management and shaping of regional food systems related to public health strategies. Public access to PUFA-enriched meat products is very unevenly distributed between highly urbanised regions and peripheral rural areas. Such disparities in the availability of affordable and healthy meat may have health implications for people living in a given geographic or territorial area [51,57,61,62,98]. The literature data indicate that there is a positive correlation between the consumption of foods that are high in omega-3 fatty acids with social groups of higher economic status benefiting from their health-promoting properties [40,61,62,63,64,99,100,101]. Reducing these inequalities requires a new approach that combines innovations in animal nutrition with the dynamic development of a given region and its health policies.

## 2. Results

### 2.1. Modulation of Cleaved Abasic Sites in the Liver of Newborn Piglets (First Day of Life) Following Maternal Oil Supplementation During Pregnancy

**Figure 1:**
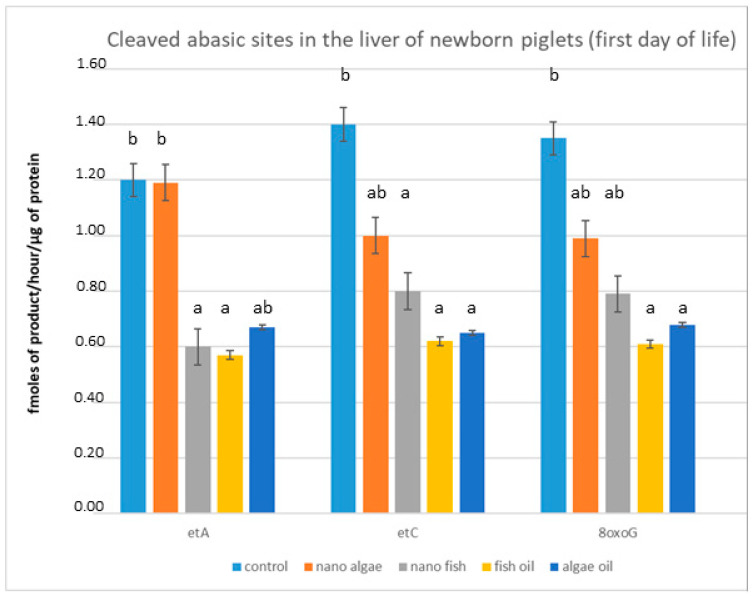
Modulation of cleaved abasic sites in the liver (fmol ethenoadducts/ug protein/hour) of newborn piglets (first day of life) from gilts fed diets supplemented with fish oil, algal oil, nanoparticles of algal oil, or nanoparticles of fish oil during pregnancy. εA, ethenoadenine; εC, ethenocytosine; 8oxoG, 8-oxoguanine. Bars labelled with different letters (a, b) are significantly different (*p* < 0.01); bars labelled “ab” do not differ significantly from bars labelled “a” or “b”.

We assessed the effect of maternal oil supplementation on the ability of livers extracted from newborn piglets (first day of life) to cleave abasic sites (Figure 1). The degree of cleaved abasic sites (εA, εC, 8oxoG) indicated that DNA repair enzyme activity in the livers of newborn piglets from gilts supplemented during pregnancy with fish oil (both forms) and algal oil was significantly lower (*p* < 0.01) compared to piglets from the control group. The greatest differences were recorded in piglets born to mothers with fish oil-inclusive diets, with changes in activity exceeding 50% for all measured enzymes. Supplementation with fish oil in nanoparticle form reduced enzyme activity by 47.1% for εA, 43.5% for εC, and 39.3% for 8oxoG. In piglets from gilts supplemented with algal oil, liver enzyme activity decreased by 50.4% for εC, 48% for 8oxoG, and 39% for εA. Algal oil in nanoparticle form did not affect εA cleavage, but activity of εC and 8oxoG was reduced by approximately 27% and 26%, respectively; these differences were statistically significant (Figure 1).

### 2.2. Modulation of Gene Expression in the Liver of Newborn Piglets (First Day of Life) Following Maternal Oil Supplementation During Pregnancy

**Figure 2:**
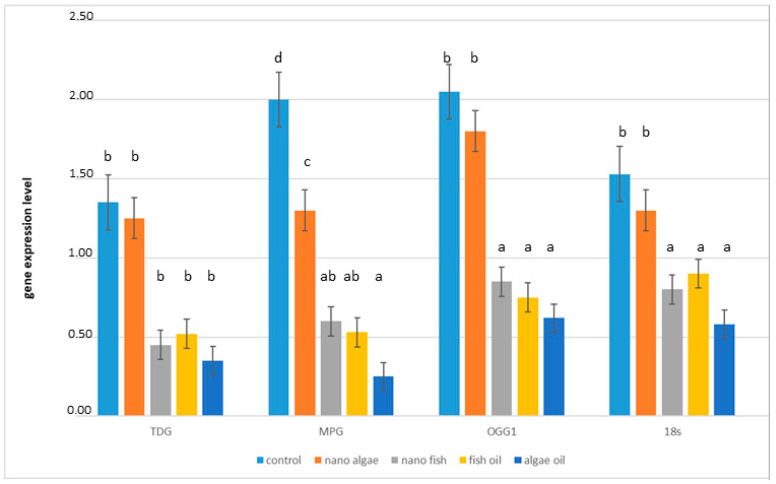
*TDG*, *MPG*, and *OGG1* mRNA expression in the liver of newborn piglets (first day of life) from gilts fed diets supplemented with fish oil, algal oil, nanoparticles of algal oil, or nanoparticles of fish oil during pregnancy. *TDG*, thymine-DNA glycosylase gene; *MPG*, N-methylpurine DNA glycosylase gene; *OGG1*, 8-Oxoguanine glycosylase gene. Values are normalised to 18S rRNA. Bars labelled with different letters (a–d) are significantly different (*p* < 0.01); bars labelled as “ab” do not differ significantly from those marked a or b.

Expression of all DNA glycosylase transcripts (TDG, MPG, OGG1) was detected in all liver samples. The relative abundance of these transcripts in all experimental groups is presented in (Figure 2). Compared to piglets from the control group, TDG mRNA expression was lower in piglets from mothers supplemented with fish oil (both forms) (mean 61.4%; *p* < 0.01). Piglets from mothers supplemented with natural algal oil showed an even greater reduction in TDG expression (71.6%; *p* < 0.01). In contrast, TDG expression in piglets from mothers supplemented with algal oil in the form of nanoparticles did not differ from the control group. For MPG mRNA expression, the largest decrease was observed in piglets from the algal oil group (85.8%, *p* < 0.01) followed by piglets from the fish oil and nano-fish groups (70.6%; *p* < 0.01), while the lowest decrease was detected in the nano-algal group (42.7%; *p* < 0.01). The expression of OGG1 mRNA was significantly reduced in piglets from the fish oil, algal oil, and nano-fish treatments (64.4%; *p* < 0.01), whereas expression in the nano-algal group did not differ from the control group. The reference gene (18S) also showed reduced expression in piglets from mothers supplemented with natural algal oil or either form of fish oil (54.3%; *p* < 0.01), while expression in the nano-algal group did not differ significantly from the control group.

### 2.3. Modulation of FPG Protein Activity in the Liver of Newborn Piglets (First Day of Life) Following Maternal Oil Supplementation During Pregnancy

**Figure 3:**
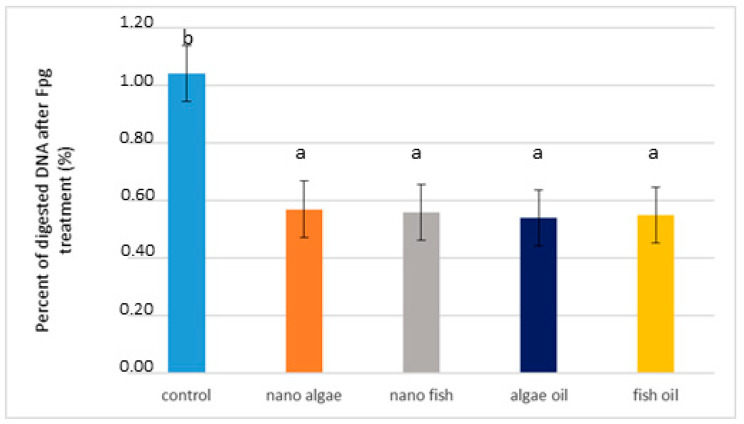
Percentage of digested DNA from liver of newborn piglets after FPG treatment (first day of life) from gilts fed diets supplemented with fish oil, algal oil, nanoparticles of algal oil, or nanoparticles of fish during pregnancy. Bars labelled with different letters (a, b) are significantly different (*p* < 0.01).

Based on the results of the nicking assay and real-time PCR, genomic DNA was isolated from liver tissue and digested with FPG, which is a bifunctional DNA repair glycosylase that serves as a marker of oxidative stress (Figure 3 and Table 1). Results showed that structural changes in genomic DNA from piglets of oil-supplemented gilts were nearly two times lower than those of the control piglets (average 0.53% vs. 0.96%, respectively; *p* < 0.01), (Table 1).

The experimental results obtained include biochemical and physiological indicators in newborn piglets. They may also have broader applications in larger food areas. Reduction in oxidative stress through the activation of DNA repair systems in piglets from gilts fed diets rich in omega-3 fatty acids suggests a very promising potential for producing meat with enhanced health-promoting parameters.

The context of regional equality is also extremely important, as areas with better-developed logistics and farmer education, as well as local government support, are more willing to adopt new innovations in animal nutrition than less developed areas, and these factors significantly deepen spatial disparities in the acquisition of functional food.

## 3. Discussion

Maternal diet is a major external factor influencing foetal development. The cells of the developing offspring are highly sensitive to maternal nutrition due to multiple cell divisions occurring at this stage [17,24,25,26,36,43,44,45,46]. Nutrition of pregnant females affects the structural and functional development, metabolism, and functions of specific cells, tissues, and systems, producing lasting effects that persist after birth [65,66,67]. These influences extend beyond growth and general health, such as birth weight, and also affect the nervous, skeletal, and circulatory systems, as well as the incidence of cancer and metabolic disorders, including obesity, in the offspring.

Adverse health events can result from DNA damage occurring even during the foetal stage of development [40,61,64]. Oxidative stress is a key factor that influences DNA integrity [1]. It arises from an imbalance between the generation of reactive oxygen species (ROS), reactive nitrogen species (RNS), and the availability of antioxidant defences [38]. Both ROS and RNS are also produced during normal cellular metabolism and can affect transcription, as well as directly inactivate DNA repair enzymes through oxidation and nitration, respectively [2,3,4]. The balance between oxidants and antioxidants is therefore crucial for preventing oxidative stress and its adverse effects on DNA repair. Oxidative damage is often a contributing mechanism of underlying liver dysfunction and genomic DNA impairment [68,69,70]. One of the major antioxidant defences against DNA damage is the base excision repair (BER) pathway [13,21,63]. It is a key DNA repair system that maintains genome stability and has been shown to prevent premature ageing, neurodegenerative diseases, and cancer [4,8,10,22,71]. Factors released during oxidative stress (e.g., 4-hydroxynonenal, and malondialdehyde) can reduce BER efficiency [72,73]. Oxidative stress is more readily induced in neonatal piglets and other mammals due to their underdeveloped immune systems [16,47,48,49,50,51,52]. Therefore, the present study focused on the effect of long-chain n-3 PUFAs with anti-inflammatory and antioxidant properties in the diet of pregnant gilts on the activity of DNA repair enzymes of the BER system, their gene expression, and FPG enzyme activity as a marker of oxidative stress in their newborns. The study specifically examined DNA lesions containing oxidatively modified guanine (8-oxoguanine, 8oxoG), adenine (ethenoadenine, εA) and cytosine (ethenocytosine, εC), which are dominant and highly mutagenic oxidative lesions [36,37,47,52,53,68,70]. The experiment also involved the study of the enzymes responsible for repairing these damages and their crucial roles in the BER pathway, along with the expression of genes that encode them. Enzymes from the aforementioned pathway show substrate specificity, efficiently removing particular types of damaged nucleobases from the DNA strand. The main enzymes involved are formamidopirymidyno-DNA glycosylase (FPG), which removes 8oxoG; thymine-DNA glycosylase (TDG), which removes ethenocytosine (εC); and N-methylpurine DNA glycosylase (MPG), which removes ethenoadenine (εA) [70]. FPG is particularly important, because it removes a wide spectrum of oxidised and alkylated bases from double-stranded DNA [4,5,6,7,8,9,10,11,74]. These enzymes are encoded by the following genes: OGG1 codes for FPG, TDG codes for TDG, and MPG codes for MPG [4,5,6,7,8,9,10,11,74]. The present study assumed that supplementation of pregnant gilts with long-chain n-3 PUFAs would influence BER enzyme activity and oxidative stress levels in their offspring. To our knowledge, this is the first study to address this issue in newborn piglets [51].

The results of the present study demonstrated that dietary supplementation of pregnant gilts with oils rich in long-chain n-3 PUFAs affected the activity of enzymes involved in the BER pathway in the livers of their newborn piglets. A lower proportion of all investigated modified bases (εA, εC, 8oxoG) found in the offspring of gilts with supplemented diets indicated that both fish and algal oils protected against occurrence of oxidative stress, significantly reducing DNA damage (mutations) and consequently reducing the activity of enzymes responsible for their removal. The greatest effect was observed in piglets from mothers fed diets containing fish oil (both natural and nanoparticle forms) and natural algal oil. Surprisingly, supplementation with nano-encapsulated algal oil did not affect the activity of the enzyme removing εA. The proportions of other DNA lesions (εC and 8oxoG) were only slightly reduced, indicating that nano-encapsulated algal oil had a limited effect on the activity of enzymes repairing these damages. These findings suggest that long-chain fatty acids crossing the placental barrier [10,72,73,74,75,76,77,96,97,98,99,100,101] not only influence structural and functional development, but also limit oxidative stress, likely by reducing the levels of ROS, RNS, and their harmful by-products in both developing foetuses and newborns. This, in turn, probably contributes to a reduction in DNA damage, and thus the decreased engagement of the BER system during repair. No data are available on the effects of supplementing pregnant females with long-chain n-3 PUFAs on BER system efficiency. However, the current literature on postnatal n-3 PUFA supplementation suggests that the reduced levels of DNA damage observed in the current study (mutated bases: εA, εC, and 8oxoG) were likely due to the anti-inflammatory properties of n-3 PUFAs. Dietary n-3 PUFA supplementation can reduce inflammation either by disrupting pro-inflammatory eicosanoid generation from arachidonic acid (AA) derivatives or by promoting the generation of anti-inflammatory forms from eicosapentaenoic acid (EPA) derivatives [78,79,80,81,96,97,98,99,100,101,102,103,104,105,106,107,108,109,110], thereby reducing pregnancy disorders and positively affecting foetal development. Contrary to the present findings, other studies reported that feeding growing rats a diet containing α-linolenic acid from rapeseed oil (ALA, C18:3 n-3), or supplementing pregnant sows with ALA provided from flaxseed, did not significantly protect against oxidative DNA damage in the colons of growing rats [80,81,82,83,84] or newborn piglets [48]. However, the same authors later reported [49] that similar dietary treatments in pregnant sows resulted in reduced 8oxoG contents in the hippocampi of newborn piglets. ALA is a precursor of longer-chain n-3 PUFAs such as EPA, whereas linoleic acid (LA, C18:2n-6) is a precursor of longer-chain n-6 PUFAs such as AA. These acids compete for the same elongase and desaturase enzymes involved in their conversion [50]. Depending on the relative abundance of each precursor, the conversion will favour either long-chain n-3 or n-6 PUFAs. A high LA-to-ALA ratio has been demonstrated to increase both the content of thiobarbituric acid reactive substances (TBARS)—a marker of oxidative stress [51]—and the expression of antioxidant enzymes [1,2,65,81], as pro-inflammatory substances are formed from AA, while anti-inflammatory substances arise from EPA conversion. Therefore, discrepancies between the present results and those of other authors may reflect differences in the metabolic activity of the liver, colon, and hippocampus. Moreover, the conversion of ALA to EPA, from which anti-inflammatory factors such as prostaglandins and cytokines are derived, is inefficient. Reported conversion efficiencies are less than 5% [80,85] or even below 1% [80,85], and the process is energetically demanding, making the dietary intake of preformed EPA more effective [80]. Consequently, the direct administration of EPA-rich oils used in this study likely stimulated the production of anti-inflammatory factors, thereby reducing oxidative stress and inflammation [85], and consequently diminishing DNA damage [3,4,5,6,7,8,9,10,11,18,22,96,97,98,99,100,101].

The literature shows that long-chain n-3 PUFAs reduce inflammation by competing with arachidonic acid for cyclooxygenase and lipoxygenase enzymes, which lowers the production of pro-inflammatory eicosanoids. They also inhibit cytokine generation, modulate nitric oxide synthesis, increase membrane fluidity, alter cell adhesion molecule expression, and reduce NF-κB activity [22]. Additionally, these fatty acids suppress lipogenesis and promote the production of protective resolving mediators, which reduces inflammation [25,36]. A recent study [76,80,85] demonstrated that long-chain omega-3 fatty acids (EPA and DHA) are used as substrates to form specialised pro-resolving mediators (SPMs), which are potent immune-protective molecules that actively reprogramme immune responses, control leukocyte trafficking, and counter-regulate the production of inflammatory mediators.

No previous studies have examined the relationship between maternal n-3 PUFA supplementation in pregnant gilts and mRNA expression of specific BER pathway enzymes in foetal or newborn organs, such as the liver. The present findings are therefore likely the first to address this question. Among oxidative DNA lesions, 8oxoG, a guanine oxidation product, is considered one of the most important biomarkers of oxidative DNA damage, as it is the most easily oxidised DNA base [13,21,77]. OOG1 is the main glycosylase that recognises and excises 8oxoG from duplex DNA [1,2,3,4,5,6,7,8,9,10,11,12,25,51]. Moreover, FPG also cleaves and excises 8oxoG lesions [1,2,3,4,5,6,7,8,9,10,11,12,51]. Hence, OGG1, MPG, TDG, and FPG play essential roles in maintaining genomic integrity in the liver of newborn piglets by eliminating DNA damage. In the presented study, gene transcripts of all examined DNA glycosylases (TDG, MPG, OGG1) were detected in all liver samples from the piglets. Supplementation of pregnant gilts with fish oils, regardless of the form they were administered in, reduced the relative gene expression of all analysed DNA glycosylases. However, regarding algal oil, only its natural form produced a similar effect. Unexpectedly, supplementation with algal oil nanoparticles did not reduce the expression of these genes, except for *MPG* mRNA. Results from the nicking assay and real-time PCR further demonstrated that in piglets from oil-supplemented gilts, bifunctional glycosylases—such as FPG, which has high affinity for 8oxoG—digested isolated genomic DNA from the livers of piglets at almost two-fold lower intensity when compared to control piglets. This means that the occurrence of oxidative stress, marked by the presence of 8oxoG, εA, and εC [14,51,77] was twice as low in these groups.

The use of n-3 polyunsaturated fatty acids in animal production can be considered a sustainable agrotechnological innovation. Its application in a given population area depends on the infrastructure of a given region based on farmers’ knowledge and the support of local government institutions. Economic tools such as Rogers’ innovation diffusion theory and geographically weighted regression (GWR) models are also helpful in spatial planning, offering a valuable framework for understanding how specific innovations can spread within a given area [51,66,87,96,97,98,99,100,101,102,103,104,105,106,107]. Basic local infrastructure and support from local governments in the form of economic incentives strongly stimulate the scope of change in a given area. At the same time, health-promoting socio–spatial support for the consumption of meat enriched with n-3 omega PUFAs should be permanently embedded in local food systems (LFSs) to strengthen food sovereignty based on health-promoting characteristics [40,61,62,63,64]. Incorporating PUFA-enriched products into local government programmes that promote healthy eating would be an important health-promoting measure for a nutritionally vulnerable population in a given territory. Efficiently produced food with rich, health-promoting properties is crucial for the production performance of farm animals, including sows. Sows with high genetic potential and large litters can successfully raise their offspring only under optimal and nutritionally stable geo- and micro-environmental conditions. These conditions encompass all key parameters related to pregnancy, parturition, and the rearing of young piglets [101,102,103,104,105,106,107].

The unexpectedly stronger protective effect of long-chain n-3 PUFAs from natural oils, resulting in reduced DNA damage and lower expression of DNA repair glycosylase genes may be due to their efficient incorporation into phospholipid membranes, where they inhibit pro-inflammatory eicosanoid production and reduce immune cell activation and pro-inflammatory cytokine release [25,45]. In turn, the lower efficacy of nanoforms in used oils could have resulted from the following reasons: (1) In theory, nanoforms are supposed to increase lipid bioavailability, but in practice, nanoemulsions can alter the absorption pathway of fatty acids; for example, they can bypass the classic mechanisms of micelle and chylomicron formation. The sows’ (and piglets’) bodies are not adapted to absorbing fat in this form, causing some of the substance to be metabolised faster or excreted [67,71,81,82,83,84,85]. (2) Nanoforms of oils could exhibit lower biological stability in the gastrointestinal tract: Nanoemulsions or nanocapsules may be more sensitive to pH and digestive enzymes, as they break down prematurely in the stomach, and fatty acids may oxidise more quickly after being released from the nanocarrier. Natural oils (e.g., fish) have their own antioxidants (vitamin E, astaxanthin), which protect omega-3 fatty acids from degradation [28,52,96,97,102]. (3) Natural fish or algae oils contain phospholipids, tocopherols, carotenoids, and sterols, which support lipid absorption and stability. In nanoparticle preparations, these compounds are removed during purification and homogenisation, reducing the bioavailability of the main components (EPA, DHA) [80]. (4) Many nanoparticle preparations are produced using high-pressure homogenization or sonication, which leads to partial oxidation of fatty acids, changes in the cis–trans configuration of some double bonds, and loss of DHA/EPA biological activity. Ultimately, such a preparation may contain fewer active fatty acids than a classic oil [71,78,83,85]. (5) Due to the large surface area of the particles, some fatty acids are adsorbed to the surface of the carrier matrix rather than reaching the bloodstream [83,85]. (6) Nanoparticles, due to their physical properties, tend to agglomerate or aggregate, which may alter their physiochemical properties, reactivity, fate, transport, and biological interactions, including bioavailability and absorption [86,87,88]. This feature may cause nanoparticles to be recognised as foreign bodies and therefore eliminated by the immune system (macrophages, enterocytes), significantly reducing their effectiveness.

Thus, from the reasons mentioned above, the long-chain n-3 PUFAs offered in this form may be less efficiently absorbed, leading to lower transfer across the placental barrier when compared with the natural form [17,25,36,44,45,87]. This reduced availability could explain the weaker protection against oxidative stress observed in the offspring of gilts supplemented with nanoparticle oils [28,30,42]. Consequently, a slightly higher activity of DNA repair glycosides and their encoding genes may be required in these piglets. Additional in-depth research is needed to confirm these hypotheses [89].

The geographic implications of this type of research determine the importance of food policy strategies in a given geographical and territorial area. This, in turn, addresses spatial inequalities in the health-promoting properties of functional foods, such as pork enriched with polyunsaturated fatty acids [90,91,92,93,94,95,96,97,98,99,100,101,102,103,104,105,106,107]. Promoting these products as part of public health education can significantly mitigate socio–territorial disparities. However, the use of functional foods depends on the regional policies of a given local government regarding its adaptability, while implementing new technological and economic factors that are rich in know-how [90,91,92]. Therefore, nutritional innovations in animal production should take into account territorial accessibility and its specific potential [101,102,103,104,105,106,107]. The following aspects should especially be considered: (1) Variability in oil composition and quality: The fatty acid composition of fish and algae oils can vary significantly, depending on the species of fish or algae, the method of oil extraction and purification, and the storage method (oxidation). This can result in inconsistent results not only in research, but also in breeding practice [99]. (2) Risk of oxidation and deterioration of feed quality: Oils rich in DHA and EPA are highly susceptible to oxidation, which leads to the formation of toxic compounds (e.g., aldehydes), reduced feed palatability, and decreased feed intake by sows. The use of antioxidants (vitamin E, tocopherols) and monitoring of storage conditions are necessary [80]. (3) Impact on the taste and aroma of sows’ milk: Excess fish oil can alter the flavour and aroma profile of milk, which can reduce its acceptance by piglets, influencing their milk intake and weight gain [96,97,102]. (4) Uneven biological responses: The effect of omega-3 fatty acids on the immune system, brain development, and lipid metabolism in piglets is dependent on the dose, duration of supplementation, and animal health, and varies between litters and even between piglets from the same litter [102]. (5) High supplementation costs: Algal oils are more expensive than vegetable or fish oils, and their use on a larger production scale increases feed costs and requires a cost-effectiveness analysis (whether the resulting health and growth benefits justify the cost) [99,101,103,108]. (6) Types of supplementations: Individual supplementation of sows is difficult to perform in common pig breeding. In turn, the addition of oils rich in long-chain PUFAs to diets can cause technological limitations. A high fat content in the mixture may reduce pellet stability, hinder the even mixing of ingredients, and require special equipment (mixers, coaters). (7) The lack of clear recommendations regarding optimal DHA/EPA doses for lactating sows and the ideal timing for supplementation.

### Limitations of the Study

We acknowledge several limitations of the present study. A significant constraint is that the analysis was conducted only on foetal liver tissue. This narrow focus resulted from the novel nature of the research question and the considerable time and labour required to conduct the animal experiment and biochemical analyses. Additional assessments in maternal tissues (e.g., blood, liver), placenta, and umbilical cord blood would have provided a more comprehensive picture of the effects of the experimental factors on both the pregnant mother and the developing foetuses. Similarly, performing measurements in a larger number of tissues in both foetuses and sows would provide a broader picture of the influence of the experimental factor administered to the mother (DHA) on the determined traits in the offspring due to possible differences in the absorption of long-chain PUFAs into the tissues of individual organs. However, this would require increasing the number of pregnant gilts per treatment group, which was not feasible within the budget constraints of the MINIATURE grant (2022/06/X/NZ9/00325), a funding programme designed for early-career researchers with limited resources. Increasing the number of sows in the feeding groups receiving specific experimental factors (type and form of long-chain fatty acid administration) would undoubtedly have a positive impact on the statistical power of the results. Furthermore, this would allow for the observation of differences in response not only between piglets from sows in specific feeding groups, but also potential differences between sows in specific feeding groups. Increasing the spectrum of analyses performed (e.g., ROS, oxidative stress markers, antioxidant enzyme activity, and other indicators related to oxidative stress) in both piglets and their mothers would provide a broader picture of the type and form of LC PUFA fed, and their influence on the course of oxidative stress in pregnant mothers, and would also allow for a more detailed understanding of the mechanisms regulating its course in developing foetuses. Addressing these limitations should be considered when interpreting the findings. In our view, the results are promising, but given the limitations, they should be regarded as preliminary. Future studies that resolve or eliminate these limitations will be essential to a better understanding of the mechanisms of action in long-chain n-3 PUFAs and the transmission of their beneficial effects from mother to foetus.

## 4. Materials and Methods

The experiment was conducted in the accredited Large Animal Models Laboratory (AAALAC International) located at the Kielanowski Institute of Animal Physiology and Nutrition, Polish Academy of Sciences. All procedures complied with the Polish Animal Protection Act and were approved by the II Local Ethics Committee on Animal Experimentation of Warsaw University of Life Sciences, SGGW, Warsaw, Poland (Resolution WAW2/040/2023). The trial was designed to minimise animal use in accordance with the 3Rs principles (replacement, reduction and refinement). This preliminary pilot experiment involved five pregnant gilts in their first gestation. The results are considered preliminary and will be utilised to design a larger study to be submitted for funding to the Polish National Science Centre in 2026.

### 4.1. Animals Housing and Feeding

**Scheme 1:**
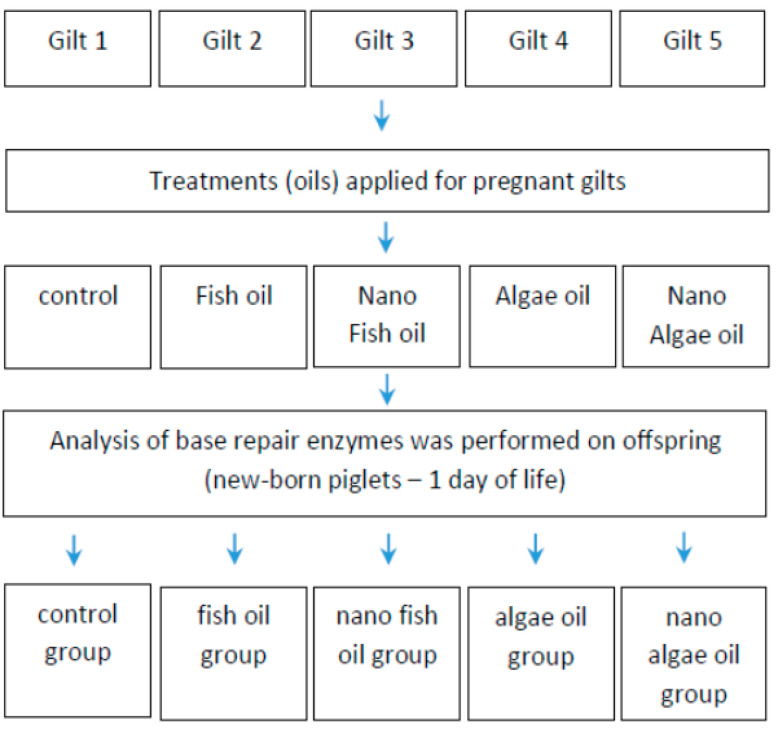
Experimental design.

The study involved five gilts (first pregnancy), each supplemented with a specific source of long-chain fatty acids during gestation, and their offspring. The research hypothesis was tested on the piglets (Scheme 1), with liver tissue samples collected for analysis.

Liver samples were collected from newborn piglets from each gilt for analysis. The pregnant gilts (Polish Landrace) were purchased from the Animal Breeding Centre in Chodeczek, Poland. After pregnancy confirmation, the animals were transported to the Laboratory of Large Animal Models at our Institute. Following an adaptation period, gilts were fed a standard diet with nutritional compositions adjusted to their gestational stage (early or late pregnancy) in accordance with NRC (2012) guidelines. Both diets were balanced for energy, protein, amino acids, and vitamins (Table 1).

After pregnancy confirmation, each gilt received a different fat supplement: fish oil, algal oil, fish oil nanoparticles, or algal oil nanoparticles. One gilt did not receive any supplement containing long-chain fatty acids and was used as the control. Fish and algal oils (in both forms) were added individually to the feed directly before feeding (Table 2).

Due to the lack of dietary recommendations for docosahexaenoic acid (DHA, C22:6 n-3) intake in pregnant sows, the daily dose was estimated based on WHO recommendations for pregnant women. Daily DHA and EPA (combined omega-3 fatty acids) intake for adults is generally 250–500 mg. For pregnant and breastfeeding women, 200 mg of DHA is recommended daily, with higher intakes of 400–600 mg or even up to 1000 mg in cases of low fish consumption or risk of preterm birth (Karol Marcinkowski University of Medical Sciences, Poznań, Poland, personal communication). Accordingly, the daily dose of natural oils and their nanoparticle forms was calculated by taking into account the multiple pregnancies in sows and the fact that the study was conducted on gilts in their first pregnancy (young females still growing). It was assumed that the expected litter size (number of developing foetuses) would be 10 piglets. Thus, each gilt received a total of 3100 mg of DHA per day, i.e., 600 mg for the gilt and 2500 mg for the developing foetus (10 foetuses × 250 mg per foetus).

Nanoparticles of fish and algal oils were prepared as described by Moghadam et al. [28]. The fish and algal oils used in the study were purchased from (NORSAN Poland LLC, Szczecin, Poland) a certified producer of oils from Arctic cod and algae. The composition of the oils is summarised in (Table 3).

Until day 90 of pregnancy, gilts were kept in individual pens (2.75 m^2^), and from day 90, they were kept in farrowing pens (5.5 m^2^). Each pen, both individual and farrowing, was equipped with a rubber mat, a feeder, a nipple drinker, central heating, and air conditioning systems (Fancom, model ISM0.12; Fancom BV, Panningen, The Netherlands) These systems allowed the animals to be maintained under thermoneutral conditions in accordance with EC Regulation (No 1924/2006).

Piglets were euthanised within the first 24 h after birth; a solution containing sodium pentobarbital was used (Exagon, 400 mg/mL, Richter Pharma AG, Wels, Austria). The product was administered into the marginal ear vein at a dose of 0.1 mL per kg body weight, following dilution 1:1 with sterile isotonic saline (0.9% NaCl). Liver samples were collected immediately after euthanasia. Although the anticipated number of foetuses per sow was estimated at 10, the actual litter size was smaller; liver samples were therefore collected from 6 piglets per sow.

After the piglets were euthanised, the sows were slaughtered at the experimental slaughterhouse of the Large Animal Models Laboratory of the Kielanowski Institute of Animal Physiology and Nutrition, Polish Academy of Sciences. Before slaughter, the sows were electrically stunned using an STZ 3 apparatus (P.P.H. MASTER Sp. J., Solec Kujawski, Poland).

### 4.2. Characteristics of the Nanoparticles and Their Systems

The nanoparticles were spherical, 31 nm in size, which ensured stable physicochemical and biological properties and enabled penetration into cells and tissues, a crucial feature for biomedical applications. The mean absolute zeta potential of the nanoparticles exceeded +40 mV, indicating strong electrostatic stability in solution (dispersion) and a low risk of particle aggregation. Zeta potential was measured using a Litesizer DLS 701 Dynamic Light Scattering Instrument (Anton-Paar, Graz, Austria). Dispersion stability was maintained for up to 14 days, reflecting strong interparticle repulsion that prevented aggregation and sedimentation. This property supports potential applications in nanomedicine, including drug delivery, transport, and bioavailability, as well as in the food industry. Encapsulation efficiency was 93%, achieved using nanoliposome technology that combined physical and chemical methods [65]. Nanoliposome encapsulation was applied to improve the bioavailability and oxidative stability of fish oil and optimise its performance. The preparations were stored at 4 °C in the dark to prevent degradation.

### 4.3. DNA Repair Activity Assay

**Scheme 2:**
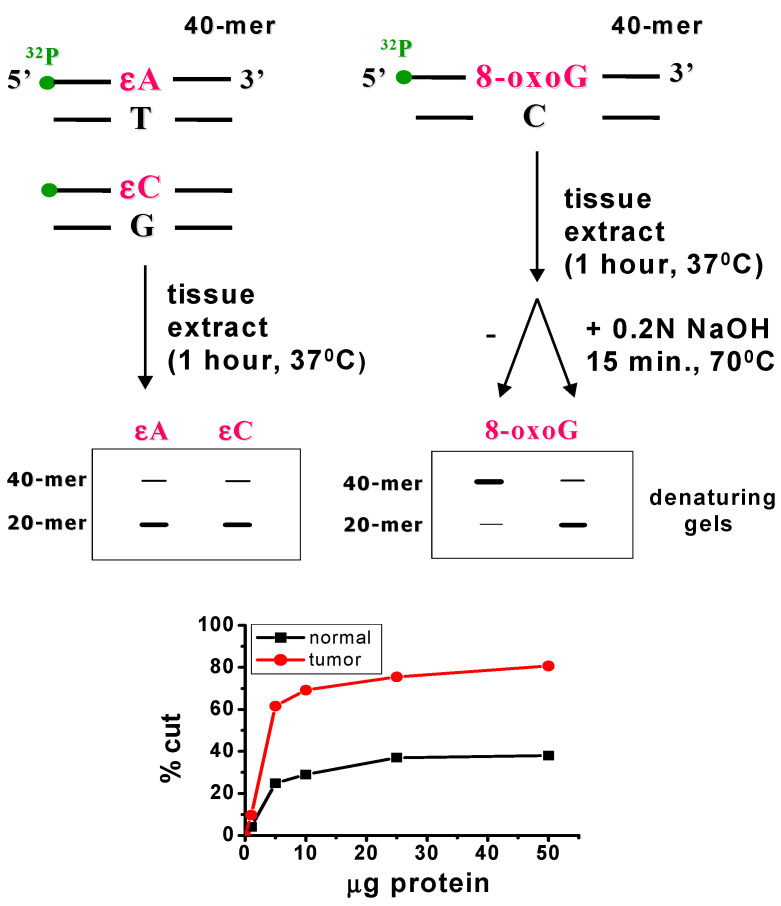
Principle of the nicking assay method.

Liver samples were analysed using the nicking assay method (Scheme 2) [10,11]. Determinations were carried out in three independent replicates, with a separate extraction of protein from colon tissue performed for each animal. Enzyme activity was calculated from the initial rates obtained under conditions of minimal non-specific inhibition, e.g., competition from proteins binding to the same DNA damage. Briefly, oligonucleotides (40-mers) containing a single 8-oxo-guanine or an apurinic/apyrimidinic (AP) site at position 20 in the sequence 5′-d (GCTACCTACCTAGCGACCTXCGACTGTCCCACTGCTCGAA)-3′ (Eurogentec Herstal, Seraing, Belgium or MetaBion, Planegg, Germany), where X denotes either 8-oxo-guanine or an AP-site, were ^32^P-labelled at the 5′-end and purified using Micro-Bio-Spin-30 (Bio-Rad, Warsaw, Poland) chromatography columns according to the manufacturer’s protocol (Bio-Rad, Scheme 2). The resulting data were used for statistical analysis (Figure S1, Supplementary Materials).

### 4.4. Real-Time Quantitative Reverse Transcription-Polymerase Chain Reaction (qPCR) Analysis of mRNA Abundance

Total cellular RNA was isolated and purificated from liver samples (20 mg) using TRIzol reagent (Invitrogen, Carlsbad, CA, USA) according to the manufacturer’s protocol [93,94,95,96,97,98,99,100,101,102,103,104,105,106,107,108,109,110,111]. Two micrograms of total DNase-treated RNA were used for reverse transcription with MMLV reverse transcriptase. The resulting cDNAs were amplified in a Light Cycler (Roche Diagnostics, Mannheim, Germany) using the respective pairs of oligonucleotide primers indicated in Table 3. Amplification products were detected using SYBR Green I (Roche Diagnostics) as described previously [93]. Specificity was verified by melt curve analysis and agarose gel electrophoresis. Data were analysed with Light Cycler 3.5 Software, and transcript expression was normalised to 18S rRNA (Table 4) [94,110,111].

### 4.5. Estimation of Genomic DNA Oxidative Damage from the Liver of Neborn Piglets

Genomic DNA was isolated from frozen liver samples using an AccuPrep. Genomic DNA Extraction Kit (Cat. No. K-3032R, Bioneer Company, Seoul, Republic of Korea) according to the manufacturer’s instructions (Figure S2, Supplementary Materials). Genomic DNA isolated from liver tissues in the template were analysed by digestion of the FPG protein. The standard reaction mixture (20 mL final volume) for the FPG protein contained; 10 mg genomic DNA, 0.09 mg FPG, 70 mM Hepes-KOH, pH 7.8, 1 mM EDTA, 5 mM b-mercaptoethanol, 100 mM KCl, 100 mg/mL BSA and 5% glycerol. Incubation was carried out at 37 °C for 30 min.

After cleavage of the genomic DNA with DNA glycosylases, the enzymes were removed by chloroform extraction. The DNA was further precipitated with 4 volume of cold 96% ethanol with 0.1 volume of 3 M sodium acetate, pH 5.2, kept at −20 °C overnight or at −80 °C for 2 h in order to be further analysed. The reaction products were separated on 1% agarose gels. After the reaction was completed, Stop Solution with 95% formamide was added to the tests and electrophoretic separation of reaction products was carried out. Electrophoresis was carried out in 1× concentrated TBE buffer for 0.5 h at 100–120 V (using the CONSORT 3000 V-300 mA high-voltage power supply, KK Wind Solutions (HQ), Ikast, Denmark).

### 4.6. Statistical Evaluation

Statistical analyses were performed using Statistica version 13.1 (StatSoft, Tulsa, OK, USA). Results are presented as mean values for each group. Data were analysed using one-way ANOVA, and where significant differences were detected, Student’s *t*-test was used pairwise comparisons. Statistical significance was set at *p* < 0.05, and trends approaching significance were considered at *p* < 0.1.

## 5. Conclusions

Supplementation of pregnant gilts with long-chain n-3 polyunsaturated fatty acids (PUFAs) appears to protect foetuses and neonates against oxidative stress. The results indicate that these fatty acids cross the placental barrier and may reduce DNA damage in offspring. This likely contributes to improved genome stability in piglets and their growth during the particularly challenging early postnatal period. Consequently, the increased genome stability in piglets from supplemented mothers resulted in reduced activity of BER pathway enzymes in their liver tissue, as less repair was needed. The results also demonstrated a slightly stronger protective effect of long-chain n-3 PUFAs when delivered in natural oils as opposed to in their nanoparticle forms.

Further multidisciplinary research is needed to examine how geographically specific factors influence the implementation of nutritional and health-promoting innovations in agriculture. We propose mapping regional readiness within a given area for the implementation of feed enriched with polyunsaturated fatty acids (PUFAs) using spatial indicators, including farm density through analysis of pro-desert areas and socio-demographic data; integrating spatial health indicators with supply chain modelling to guide evidence-based policy in the food and health sectors; ensuring unrestricted access to veterinary services; ensuring consumer access to PUFA-rich meat products; and incorporating spatial equity measures into nutritional programming to ensure the full foetal health benefits of dietary interventions are available to all mothers in all regions. This regional mapping provides a roadmap for sustainable innovations in sustainable livestock production and nutrition in a public health context and complements the biological research model presented in this manuscript.

## Figures and Tables

**Table 1:** DNA damage, gene expression, and FPG activity in liver samples from newborn piglets following maternal oil.

Supplementation						
Item	Control	Nano-Algae	Nano-Fish	Algae Oil	Fish Oil	S.E.
Cleaved abasic sites [fmol ethenoadducts/μg protein/h]						
εA	1.205 b	1.192 b	0.637 a	0.735 ab	0.550 a	0.126
εC	1.438 b	1.045 ab	0.812 a	0.713 a	0.675 a	0.146
8oxoG	1.352 b	1.000 ab	0.820 ab	0.702 a	0.642 a	0.143
Relative genes expression [A.U]						
TDG	1.297 b	1.090 b	0.473 a	0.368 a	0.527 a	0.099
MPG	1.993 d	1.142 c	0.608 ab	0.282 a	0.563 ab	0.176
OGG1	2.050 b	1.748 b	0.840 a	0.602 a	0.747 a	0.164
18S	1.537 b	1.297 b	0.732 a	0.528 a	0.848 a	0.106
FPG treatment [percentage of genomic DNA recognised by FPG enzyme]						
FPG	0.960 b	0.545 a	0.542 a	0.507 a	0.532 a	0.016

εA, ethenoadenine; εC, ethenocytosine; 8oxoG, 8-oxoguanine; TDG, thymine-DNA glycosylase gene; MPG, N-methylpurine DNA glycosylase gene; OGG1, 8-oxoguanine glycosylase gene; FPG, formamidopirymidyno-DNA glycosylase; 18S, 18S rRNA reference gene. Means with different letters (a–d) are significantly different (*p* < 0.01).

**Table 2:** Nutritional composition of the standard diet for gilts, adapted to physiological stage of pregnancy.

Item	Day of Pregnancy	
	Till 90	90–114
Nutritional value (per kg diet)		
Crude protein, g	145	175
Lysine, g	8.0	10.0
Methionine	3.0	3.0
Methionine + Cystine	6.0	7.0
Threonine, g	6.0	6.5
Tryptophan, g	2.0	2.0
Crude fibre	50.0	36.0
Calcium, g	8.0	9.0
Total phosphorus	6.0	6.5
Digestible phosphorus, g	2.0	3.0
Vitamin A, IU	13,000	12,500
Vitamin E, mg	100	100
Vitamin D, IU	2000	2000
Metabolisable energy, MJ	12.0	13.0

**Table 3:** Fatty acid contents of fish and algal oils used in the study.

Ingredient	Norsan Omega-3 Arktis (Per 10 mL)	Norsan Omega-3 Vegan (Per 10 mL)
Components		
Sunflower oil	-	-
Fish oil	8.8 g	-
Algae oil	-	7.0 g
Olive oil	-	2.2 g
Fatty acid composition		
SFA	1.8 g	-
MUFA	3.9 g	-
PUFA	2.3 g	4.4 g
Omega-3	2.0 g	4.0 g
EPA	750 mg	1218 mg
DPA	95 mg	314 mg
DHA	975 mg	2316 mg

**Table 4:** Oligonucleotide primers used in real-time quantitative RT-PCR.

Gene Specific Primer	Sequence (5′ to 3′)
MPG MpgF	GTCCTATCCGGCGACTTCC
MPG MpgR	CTTGTCTGGCAGGCCCTTTG C
Ogg1F	CTCAGAAATTCCAAGGTGTTC
Ogg1R	CCGCTCCACATGCCAGTG
18SpfF	ATCCTTCGATGTCGGCTCTT
18SpfR	ACTAACCTGTCTCAGACGGTC
TDGpeF	TAATGGGCAGTGATGACCC
TDGpeR	TGCAGCATTTAGCAGAGCTGA

## Data Availability

Data is contained within the article and Supplementary Materials. Further inquiries can be directed to the cor-responding author.

## Supplementary Materials

The following supporting information can be downloaded at https://www.mdpi.com/article/10.3390/ijms262110676/s1.

## Abbreviations

BER, base excision repair; PUFA, polyunsaturated fatty acid; TDG, thymine-DNA glycosylase gene; MPG, N-methylpurine DNA glycosylase gene; OGG1, 8-oxoguanine glycosylase gene; ROS, reactive oxygen species; RNS, reactive nitrogen species; EPA, eicosapentaenoic acid; DHA, docosahexaenoic acid; TAC, total antioxidant capacity; GPx, glutathione peroxidase; MDA, malondialdehyde; PGE2, prostaglandin E2; PGE3, prostaglandin E3; (εA)-1,N^6^, ethenoadenine; (εC), 3,N^4^-ethenocytosine; (εG), N^2^,3-ethenoguanine; (εG), 1,N^2^-ethenoguanine; 8oxoG, 8-oxoguanine; PAGE, poly acrylamide electrophoresis; qPCR, real-time quantitative reverse transcription-polymerase chain reaction; MMLV, Moloney murine leukaemia virus reverse transcriptase; FPG, formamidopirymidyno-DNA glycosylase; 18s, reference gene 18S rRNA; LA, linoleic acid; ALA, alpha linolenic acid; AA, arachidonic acid; TABRS, thiobarbituric acid reactive substances.
