# Supplementary material for: Biochemical Effects of Natural and Nanoparticle Fish and Algal Oils in Gilt Pregnancy Diets on Base Excision Repair Enzymes in Newborn Piglets—Socioeconomic Implications for Regional Pig Farming—Preliminary Results
Source: Int J Mol Sci. 2025 Nov 2;26(21):10676. doi: 10.3390/ijms262110676 (PMC12608807; doi:10.3390/ijms262110676)
Supplement: Supplementary file 1 [file ijms-26-10676-s001.zip › ijms-3922014-supplementary.pdf]

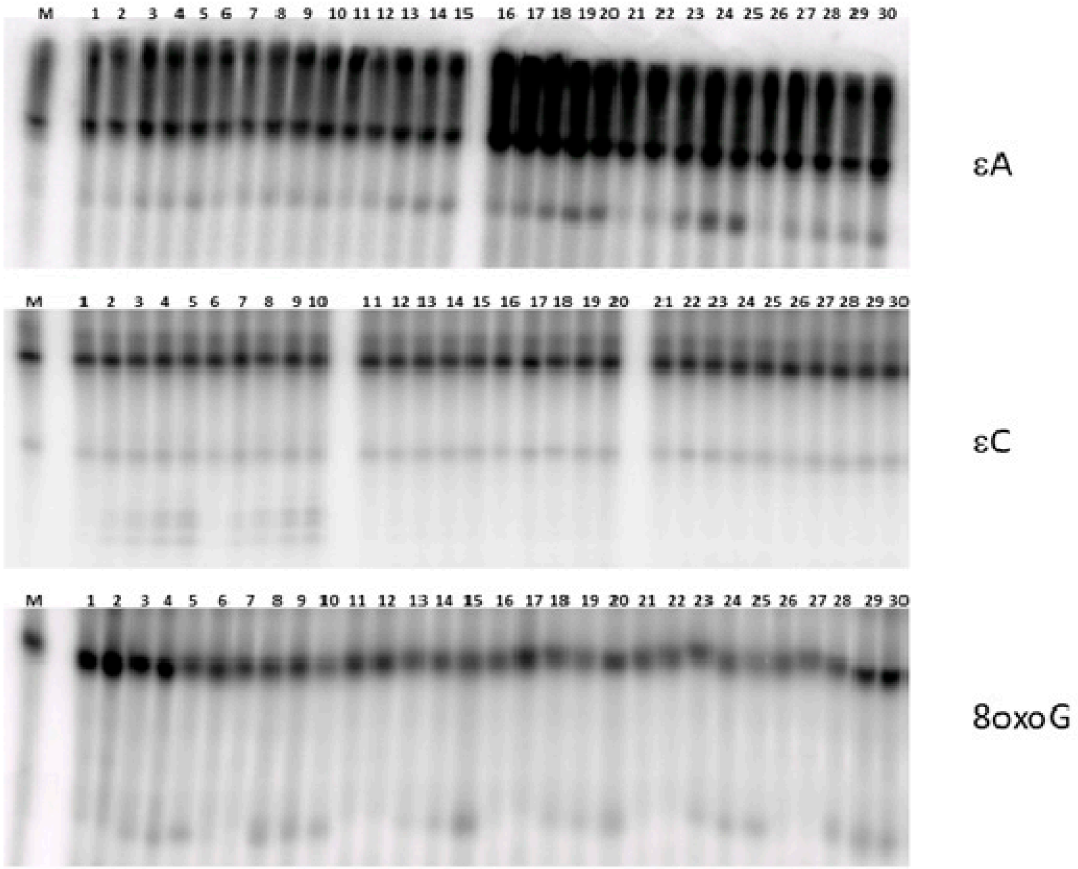

Figure S1. Exaple of polyacrylamide gels including analysed probes (newborn pigs treated with combination of oils fish or algae oil with nanoparticles and measured by nicking assay method). Samples marked from 1 to 6 after treatment sunflower oil, samples 7-12 were treatment nano algae , samples 13-18 were treatment nano fish mix, samples 19-24 were treatment algae oil, samples 25-30 were treatment fish oil.

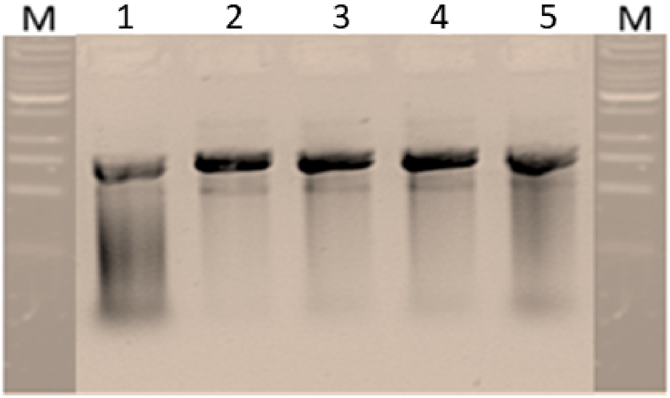

Figure S2. Exaple of agarose gels including analysed probes (newborn pigs treated with combination of oils fish or algae oil with nanoparticles and cleaved by Fpg enzyme). Samples marked from 1 to 5 1 treatment sunflower oil (as control) , samples 2 were treatment nano algae , samples 3 were treatment nano fish mix, samples 4 were treatment algae oil, samples 5 were treatment fish oil.
